# The burden of cystic fibrosis in North Africa

**DOI:** 10.3389/fgene.2023.1295008

**Published:** 2024-01-10

**Authors:** Nada El Makhzen, Houria Daimi, Laila Bouguenouch, Hugues Abriel

**Affiliations:** ^1^ Ion Channels and Channelopathies Laboratory, Institute for Biochemistry and Molecular Medicine, University of Bern, Bern, Switzerland; ^2^ Laboratory of Human Genome and Multifactorial Diseases (LR12ES07), Faculty of Pharmacy, University of Monastir, Monastir, Tunisia; ^3^ Department of Biology, Faculty of Sciences, University of Gabes, Gabès, Tunisia; ^4^ Laboratory of Medical Genetics and Oncogenetics, University Hospital Hassan II, Sidi Mohamed Ben Abdellah University, Fez, Morocco

**Keywords:** CF, CFTR, monogenic disease, rare disease, North Africa

## Abstract

**Background:** Over 200 pathogenic variants in the cystic fibrosis transmembrane conductance regulator (*CFTR*) gene are associated with cystic fibrosis (CF)—the most prevalent autosomal recessive disease globally, the p.Phe508del variant being the most commonly observed.

**Main text:** Recent epidemiological studies suggest a higher global prevalence of CF than previously thought. Nevertheless, comprehensive CF data remains extremely scarce among African populations, contributing to a significant information gap within the African healthcare system. Consequently, the underestimation of CF among children from African populations is likely. The goal of this article is to review the pathogenesis of CF and its prevalence in the countries of North Africa.

**Conclusion:** The prevalence of CF in North African countries is likely underestimated due to the complexity of the disease and the lack of a timely, proper clinical and genetic investigation that allows the early identification of CF patients and thus facilitates therapeutic recommendations. Therefore, specific genetic and epidemiological studies on African individuals showing CF symptoms should be conducted to enhance the diagnostic yield of CF in Africa.

## Introduction

Cystic fibrosis (CF) (CF; MIM# 219700) is an autosomal recessive genetic disease caused by variants in the cystic fibrosis transmembrane conductance regulator (*CFTR*; MIM *602421) gene, which affects the function of this ion channel protein to maintain chloride balance across apical membranes (Stewart and Pepper, 2017). More than 2000 variants have been identified in the *CFTR* gene, with more than 200 responsible for CF (http://www.cftr2.org/index.php); additionally, p.Phe508del is the most common variant. An epidemiological review of these pathogenic variants revealed that they are often population-specific, ranging according to country of origin and ethnicity (Bobadilla et al., 2002).

CF is the most common autosomal recessive disease in individuals of European descent and is characterized by chronic lung disease, pancreatic insufficiency, elevated sweat chloride concentration levels, and obstructive azoospermia (Gajbhiye and Gaikwad, 2017). In addition, epidemiological studies conducted over the last 2 decades have demonstrated that CF occurs more commonly than previously thought in populations of non-European descent, and the disease is now recognized in many parts of the world (Bell et al., 2020a). It is estimated that CF affects approximately 72,000 patients worldwide (Hammoudeh et al., 2021), with a rate of 1/2000 in European ancestry populations (Gajbhiye and Gaikwad, 2017), an incidence of 1 in 12,000 in South Africa’s mixed ancestry population (Carles et al., 1996; Feuillet-Fieux et al., 2004) and an incidence of 1 in 14,000 in African American black people. However, the occurrence of CF in black African populations with no European ancestry contribution is unknown (Carles et al., 1996). This variation in reported incidence arises from differences in the sampled population and the detection method used, whether it is newborn screening, newly reported cases, or calculations based on death certificates (Hamosh et al., 1998a).

This article aims to review the fundamental molecular and cellular mechanisms of CF, its prevalence in North African countries (Morocco, Algeria, Egypt, Libya, and Tunisia), and the pathogenic variants identified within these populations. We herein discuss the information gap regarding the epidemiology of the disease in these countries as well as the challenges impeding a proper CF diagnosis in North African patients. Based on the available data, we present our vision of the possible actions that can be taken to overcome these challenges and fill the knowledge gaps.

## CFTR (cystic fibrosis transmembrane conductance regulator)

### Overview of CFTR structure, function and regulation

**FIGURE 1:**
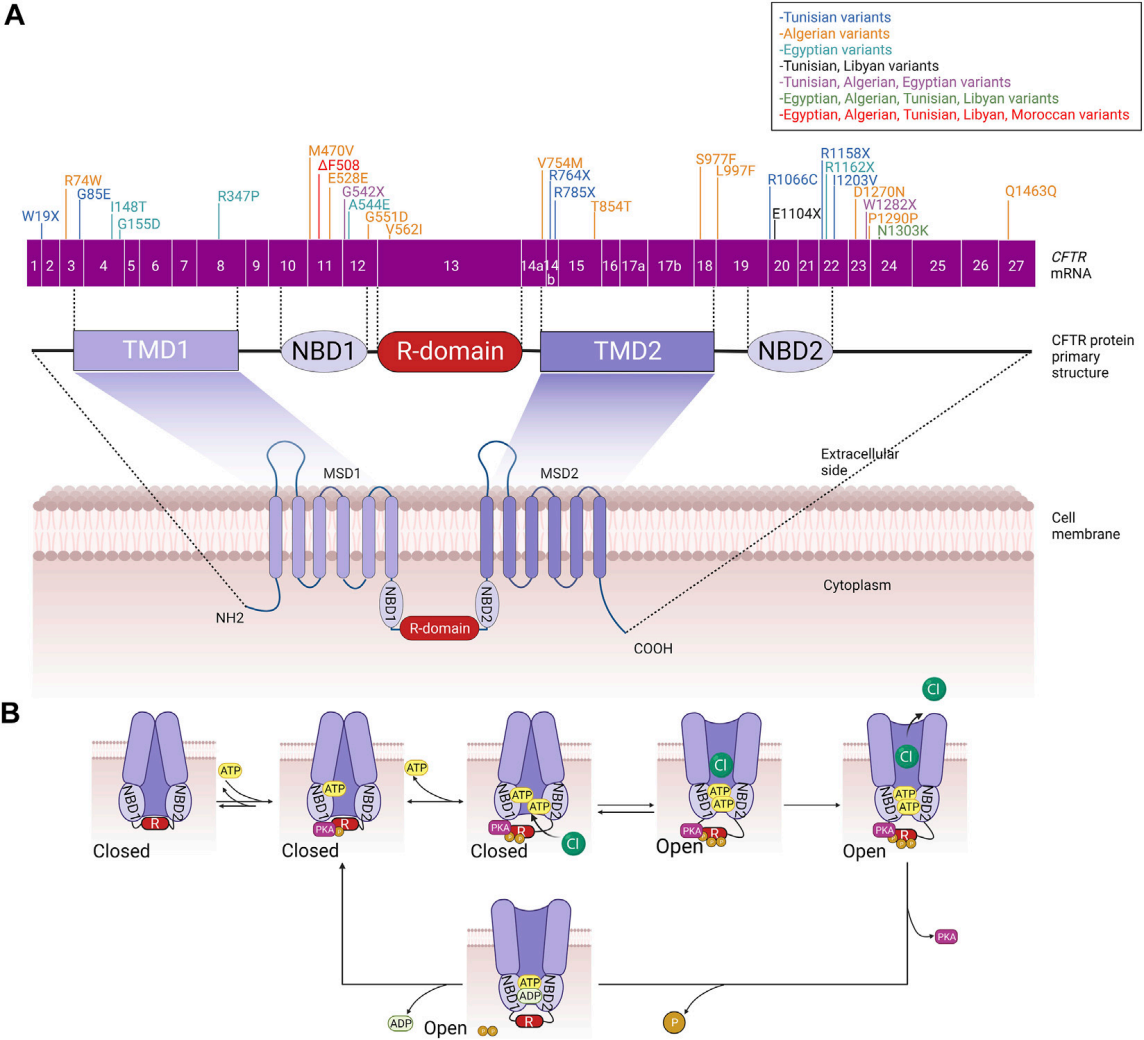
**(A)** CFTR primary and secondary structure. CFTR coding sequence variants identified in the different North African populations are included. **(B)** Schematic summary of phosphorylation-mediated CFTR gating regulation. MSD: membrane-spanning domain, TMD: transmembrane domain, NBD: Nucleotide-binding domain, R: regulatory domain.

The cystic fibrosis transmembrane conductance regulator (*CFTR*) was first cloned in 1989 (Riordan et al., 1989), which enabled studies into its structure, function, regulation, and biogenesis (Akabas, 2000). *CFTR* is localized on human chromosome 7, long arm, region q31-q32 and is approximately 250 kb in size with 27 exons (Zielenski et al., 1991). The *CFTR* gene encodes for a 1480 amino acids ABC (ATP-binding cassette) membrane transport protein (Akabas, 2000), the structure of which is mainly conserved among ABC transporters. It comprises five functional domains: two hydrophobic membrane-spanning domains (MSD1, MSD2), two hydrophilic membrane-associated domains containing nucleotide-binding domains (NBD1, NBD2), and a distinctive regulatory (R) domain containing multiple consensus sequences for phosphorylation by protein kinases A (PKA) and C (PKC) (McCarty, 2000; Kleizen et al., 2020) (Figure 1A).

Interestingly, CFTR is considered the only ABC protein that functions as an ion channel since almost all other ABC proteins act as transport ATPases. In addition, thanks to its enzymatic activity, CFTR is also the only ligand-gated channel that consumes its ligand (ATP) during the gating cycle as a molecular mechanism mediating the channel closure (Figure 1B). These two distinguishing features caught the attention of researchers in the field because, accordingly, CFTR did not seem to fit perfectly into the ABC “transporter” scheme, suggesting that CFTR is likely a result of evolution on an ABC transporter, turning it into an ion channel (Hwang and Kirk, 2013). CFTR is a channel that permits anions to flow across the membrane in either direction (absorptive or secretory). However, while chloride is the most abundant inorganic anion in the body, the CFTR pore primarily conducts Cl- and bicarbonate ions (HANRAHAN et al., 2003). CFTR is a phosphorylation-dependent epithelial Cl-channel and is expressed in the plasma membranes of several epithelial cells, including those of the kidney, gut, pancreas, sweat glands, and conducting airways, where it permits transepithelial Cl-flow (Sheppard and Welsh, 1999; McCarty, 2000). However, growing evidence indicates that CFTR is also present in the intracellular compartments such as the endosomes, lysosomes, phagosomes and mitochondria. Consequently, impairment of CFTR function leads to impairment of ion flow across the epithelial tissues and affects the proper functioning of these organelles (Lukasiak and Zajac, 2021).

Regulation of CFTR biogenesis, traffic and gating, once delivered to the plasma membrane, determines the proper activity of the channel and thus ensures the fine-tuned modulation of chloride secretion by the epithelial cells. In this context, CFTR shares with other transmembrane proteins the common biogenesis and traffic features that include mRNA transcription and alternative splicing that take place in the nucleus; translation, protein folding, and core glycosylation occurring at the endoplasmic reticulum; trafficking to and across the Golgi apparatus that includes further glycosylation and other posttranslational modifications such as phosphorylation and ubiquitination; and finally vesicular delivery to the cell surface (McClure et al., 2016). However, during the last decades and with the revolutionary involvement of structural biology in resolving protein structure and function, particular models of CFTR gating mechanisms have been proposed (Csanády et al., 2019). Almost all these models share the common feature that CFTR closure/opening is associated with an ATP binding/hydrolysis cycle at NBD1 and NBD2 and a phosphorylation/dephosphorylation cycle of the R domain. One of the suggested mechanisms that control the closed state of the CFTR channel implicates the interaction between NBD1 and the R domain. This physical interaction impedes NBD1 to dimerize with NBD2. To allow channel opening and chloride secretion, the NBD1-R domain complex is separated by the multi-sites protein kinase A (PKA)-mediated phosphorylation of the R domain, which triggers large conformational rearrangements at the TMDs that gradually attenuate these steric hindrances leading finally to the release of the R domain and allowing the head-to-tail heterodimerization of NBD1 and NBD2.

Consequently, ATP binds to the CFTR channel, leading to the opening of the channel pore and the secretion of chloride ions. ATP hydrolysis and CFTR dephosphorylation will allow the R domain to wedge between the NBDs and the channel to come back to its closed state (Hegedus et al., 2009; Corradi et al., 2015; Liu et al., 2017; Della Sala et al., 2021) (Figure 1B). Like many other ion channels, CFTR channels are also able to aggregate in dimers and form a microdomain through connection with other interacting proteins such as synaptosome-associated protein, 23 kDa (SNAP23), AMP kinase (AMPK), protein phosphatase-2A (PP2A), syntaxin-1A (SYN1A), and Munc-18a which promote channel inactivation/closure or protein kinase C (PKC), Na+/H+ exchanger regulatory factor isoform-1 (NHERF1), ezrin and receptor for activated C-kinase-1 (RACK1) that in the opposite allow the efficient PKA-mediated phosphorylation of CFTR and ultimately its activation/opening (Guggino and Stanton, 2006).

It is important to note that the CFTR protein is abnormally expressed in several types of tumour cells that originated as epithelial cells. There is a recent interest in the correlation between abnormal CFTR protein (including expression and mutations) and various cancers. For instance, a study demonstrated that CFTR was highly expressed in Ph + acute leukaemia cells, which protected and maintained the continuous activation of BCR-ABL and the canonical Wnt/β catenin signalling pathway by decreasing PP2A phosphatase activity (Yang et al., 2017). To the best of our knowledge, no direct link has been reported so far between CF and the development or progression of Ph-like acute leukaemia.

### CFTR variants

**FIGURE 2:**
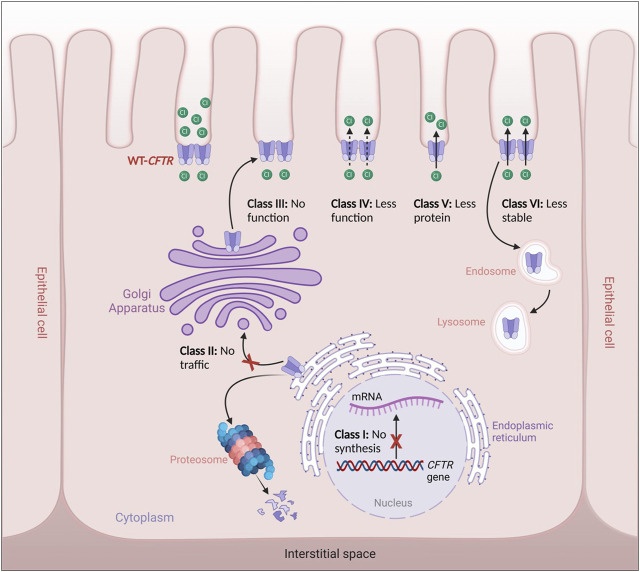
Classes of CFTR variants. Class I: defective CFTR gene transcription, Class II: defective CFTR protein trafficking from the endoplasmic reticulum to the Golgi apparatus leading thus to its degradation by the proteosomes, Class III: Defective CFTR regulation leading to non-functional CFTR channels, Class IV: Defective CFTR function leading to reduced CFTR gating, Class V: Defective CFTR processing leading to reduced cell surface expression, Class VI: Production of less stable CFTR channels leading thus to their recycling by the endosome/lysosome system.

Alteration in the CFTR protein processing or function leads to the impairment of the channel in epithelial cells, which in turn results in the accumulation of more viscous mucus, primarily in the lungs, where the most severe symptoms occur, and also in the pancreas, hepatobiliary tree, gastrointestinal tract, sweat glands, and genital apparatus. To date, there are more than 2000 variants that have been found in the *CFTR* gene. Initially, these variants were classified into five (and occasionally six) functional groups, and these class systems provide a practical foundation for identifying fundamental defects at the cellular level (Cutting, 2015). CFTR pathogenic variants are classified according to the following classes (Cant et al., 2014; Gajbhiye and Gaikwad, 2017) (Figure 2):

• Class I: CFTR manufacturing ends prematurely due to early termination of transcription, which reduces or abolishes CFTR protein synthesis. The most common class I pathogenic variants are p.Gly542Ter in Mediterranean countries, p.Arg1162Ter and p.Trp1282Ter in Ashkenazy Jews.
• Class II: There is no appropriate processing of CFTR and proteins within the cell. This class includes the common p.Phe508del variant, responsible for >90% of CF cases that have been reported thus far (Cant et al., 2014), which is translated into full-length nascent polypeptide chains but fails to fold and is consequently targeted for destruction rather than trafficked to the plasma membrane (PM).
• Class III: CFTR reaches the cell surface but does not appropriately open to transport chloride. This functional class, which includes the second most frequent pathogenic variant p.Gly551Asp, is seen in just a small number of CF patients (2%–3%).
• Class IV: CFTR reaches the PM but has lower channel conductance even when the gate is open. These are rare variants that cause disease in 2% of CF patients and are generally located in the MSDs, including p.Arg117His and p.Arg334Trp or p.Arg347Pro in MSD1
• Class V: The least prevalent functional class; it represents a completely functioning CFTR at the PM but with lower abundance due to incorrect mRNA splicing. An example of these pathogenic variants is the p.Ala455Glu variant.
• Class VI: Even though the CFTR protein functions, it is unstable at the cell surface (Gajbhiye and Gaikwad, 2017). This variant class includes the p.Gln1412Ter variant.

All CF patients have a combination of two (or more) variants (Cant et al., 2014; Bareil and Bergougnoux, 2020). The disease phenotype varies by the severity of the CFTR variants. In this regard, it has been assumed that when CF-causing pathogenic variants are coupled, they cause severe CF clinical symptoms. However, moderate or mild variations are associated with CFTR-related disorders (CFTR-RD), and such patients present diffuse bronchiectasis, pancreatitis, male infertility, and congenital bilateral absence of vas deferens). A limited number of *CFTR* variants are linked to a wide range of phenotypes, ranging from CF to CFTR-RD or from CFTR-RD to no symptoms. On the other hand, there are many genetic variants with undefined clinical significance since they are extremely rare and have not yet been functionally investigated (Bareil and Bergougnoux, 2020).

### CF diagnosis in Africa and challenges

There is a notable lack of information on CF in the populations of the African continent. Despite a limited number of reports from African communities, reports from South African blacks populations stated that many children with CF are likely misdiagnosed due to the similarities between the CF phenotype and that of phenocopic pathologies frequently characterized in Africa such as, primary protein energy malnutrition (PEM) manifesting elevated sweat chloride values, tuberculosis, recurrent lung infections, infantile diarrhoea, HIV/AIDS, failure to thrive, or a high infant mortality rate (Rodrigues et al., 1994; Padoa et al., 1999). Only after twin black African boys were identified as the first diagnosed cases of CF at Johannesburg’s Baragwanath Hospital did researchers recommend that clinicians consider CF a potential diagnosis in this ethnic group. Sadly, the first set of premature ethnic Bantu twins with CF born at the hospital succumbed to meconium ileus, with a subsequent tragic case detailed in an addendum (Hargraves et al., 1948). The first report of CF pathogenic variants in South African black patients with no known white admixture showed that 3120+1G-A is a common variant in African black people (Carles et al., 1996). While CF is considered an uncommon disease among African populations, and since screening sweat tests are hardly performed in many African hospitals and can be challenging to interpret, many clinicians do not consider CF a probable diagnosis. For example, CF was formerly diagnosed in Sudan based on history and clinical and radiological data. The sweat test first appeared in 2008 and was only available at one hospital in Khartoum (Ibrahim et al., 2014). Moreover, molecular genetic tests necessary to diagnose CF are costly and mostly unavailable in many African populations; this leads to a lack of accurate and timely CF diagnosis, leading to early complications and mortality due to insufficient medical care (Mutesa and Bours, 2009).

The scarcity of CF data in populations of non-European descent is particularly evident across Africa. Except for South Africa, the absence of patient registries for this condition means African CF patients cannot access treatment therapies based on established registry data patterns. Even though there is a vast genetic variation within African populations, their genomes have been relatively under-investigated (Van Rensburg et al., 2018). However, researchers have revealed that the clinical symptoms of CF are identical in black and non-black individuals, with the notable exception of black patients having lower nutritional status, which appears to be independent of age and genotype. Black people with CF had more severe gastrointestinal difficulties, as indicated by lower nutritional status and a higher frequency of distal intestinal obstruction syndrome (DIOS), which may be responsive to intensive dietary management. Consequently, the diagnosis should be investigated in black individuals with unexplained chronic lung disease or malabsorption symptoms (Hamosh et al., 1998b).

### Variants identified in North African countries

For this review article, we surveyed CF on the African continent using Google Scholar and PubMed, searching for the term “cystic fibrosis” alongside the names of North African countries: Morocco, Algeria, Egypt, Libya, and Tunisia. The aim was to report on the genetic screening data of North African individuals carried out within Africa. A total of 17 reports on molecular research into the cause of CF were published by clinicians and researchers from five North African countries (see Table 1).

**TABLE 1:** Summary of reported variants in North African CF patients conducted in Africa.

Variants	rs Id	North African countries in which identified	References
c.1521_1523del (p.Phe508del)	rs113993960	Egyptian, Algerian, Tunisian, Libyan, Moroccan	Shahin et al. (2016); Al-Haggar et al. (2020), Loumi et al. (1999); Loumi et al. (2008); Sediki et al. (2014), Messaoud et al. (1996); Fredj et al. (2009); Hadj Fredj et al. (2011); FREDJ et al. (2013); Boussetta et al. (2018); Hamouda et al. (2020), Ratbi et al. (2008)
c.1624G>T (p.Gly542Ter)	rs113993959	Tunisian, Algerian, Egyptian	Fredj et al. (2009); Boussetta et al. (2018), Sediki et al. (2014), Al-Haggar et al. (2020)
c.3909C>G (p.Asn1303Lys)	rs80034486	Egyptian, Algerian, Tunisian, Libyan	Shahin et al. (2016), Loumi et al. (1999); Loumi et al. (2008); Sediki et al. (2014), Loumi et al. (2008), Hadj Fredj et al. (2011)
c.3846G>A (p.Trp1282Ter)	rs77010898	Tunisian, Algerian, Egyptian	Fredj et al. (2009), Loumi et al. (2008), Shahin et al. (2016); Al-Haggar et al. (2020)
c.3310G>T (p.Glu1104Ter)	rs397508538	Tunisian, Libyan	Fredj et al. (2009), Hadj Fredj et al. (2011)
711 + 1G>T	rs77188391	Algerian, Tunisian	Loumi et al. (1999); Loumi et al. (2008); Sediki et al. (2014)
c.3808G>A (p.Asp1270Asn)	rs11971167	Algerian	Loumi et al. (2008)
c.220C>T (p.Arg74Trp)	rs115545701	Algerian	Loumi et al. (2008)
c.1680-1G>A (1812–1G→A)	rs121908794	Algerian	Loumi et al. (1999); Loumi et al. (2008)
c.1584G>A (p.Glu528 = )	rs1800095	Algerian	Loumi et al. (2008)
c.2930C>T (p.Ser977Phe)	rs141033578	Algerian	Loumi et al. (2008)
c.1652G>A (p.Gly551Asp)	rs75527207	Algerian	Sediki et al. (2014)
c.4139delC (4271delC) p.(Thr1380AsnfsX4)	rs397508680	Algerian	Loumi et al. (2008)
c.4200_4201delTG (p.Cys1400X)	rs397508695	Algerian	Loumi et al. (2008)
c.489 + 3A>G (621+3A/G)	rs377729736	Algerian	Loumi et al. (2008)
c.2260G>A (p.Val754Met)	rs150157202	Algerian	Loumi et al. (1999); Loumi et al. (2008)
c.1684G>A (p.Val562Ile)	rs1800097	Algerian	Loumi et al. (2008)
c.2051_2052delinsG (2183AA/G) (p.Lys684SerfsX38)	rs121908799	Algerian, Egyptian	Loumi et al., (2008), Shahin et al., (2016)
c.1210-12T[5] (5T)	rs1805177	Egyptian, Algerian, Moroccan	Shahin et al. (2016), Loumi et al. (1999); Loumi et al. (2008), Ratbi et al. (2008)
c.1408G>A (p.Val470Met)	rs213950	Algerian	Loumi et al. (1999); Loumi et al. (2008)
c.3870A>G (p.Pro1290 = )	rs1800130	Algerian	Loumi et al. (1999)
c.2562T>G (p.Thr854 = )	rs1042077	Algerian	Loumi et al. (1999); Loumi et al. (2008)
c.4389G>A (p.Gln1463 = )	rs1800136	Algerian	Loumi et al. (1999); Loumi et al. (2008)
c.743 + 40A>G (875 + 40A→G)	rs1800502	Algerian	Loumi et al. (1999); Loumi et al. (2008)
c.869 + 11C>T (1001 + 11C/T)	rs1800503	Algerian	Loumi et al. (2008)
c.2991G>C (p.Leu997Phe)	rs1800111	Algerian	Loumi et al. (2008)
c.3870A>G (p.Pro1290 = )	rs1800130	Algerian	Loumi et al. (2008)
c.1584 + 51_1584_61dup11 (dup1716 + 51→61)	rs397508232	Algerian	Loumi et al. (2008)
c.1679 + 5A>G (1811 + 5A>G)	rs397508264	Tunisian	Fredj et al. (2009)
c.3889dup (4016insT) (p.Ser1297fs)	rs121908808	Tunisian	Fredj et al. (2009)
4268 + 2T>G	rs1562928998	Tunisian	Fredj et al. (2009)
c.3607A>G (p.Ile1203Val)	rs75647395	Tunisian	Fredj et al. (2009)
c.3472C>T (p.Arg1158Ter)	rs79850223	Tunisian	Fredj et al. (2009)
c.2353C>T (p.Arg785Ter)	rs374946172	Tunisian	Fredj et al. (2009)
c.254G>A (p.Gly85Glu)	rs75961395	Tunisian	Boussetta et al. (2018); Hamouda et al. (2020)
c.1392 + 6_1392+7insA (1524 + 5 InsC)	-	Tunisian	Hamouda et al. (2020)
c.2290C>T (p.Arg764Ter)	rs121908810	Tunisian	Hamouda et al. (2020)
c.1545_1546delTA (1677delTA) (p.Tyr515Ter)	rs121908776	Tunisian	Hamouda et al. (2020)
del ex1-2	NC_000016.10	Tunisian	Hamouda et al. (2020)
c.3196C>T (p.Arg1066Cys)	rs78194216	Tunisian	Hamouda et al. (2020)
c.3598_3599delinsG (p.Lys1200fs)	rs1584830149	Tunisian	Hamouda et al. (2020)
c.2766del8 (p.Leu878PhefsX15)	-	Tunisian	Messaoud et al. (1996)
c.1680-1G>A (1812-1G>A)	rs121908794	Algerian	Loumi et al. (1999); Loumi et al. (2008)
c.1670delC (p.Ser557fs)	rs397508257	Libyan	Hadj Fredj et al. (2011)
c.1477_1478del (1609delCA) (p.Gln493fs)	rs121908775	Algerian	Loumi et al. (2008)
CFTRdel2,3 (21 KB) (p.Ser18ArgfsX16)	VCV000066105.9	Algerian, Egyptian	Loumi et al. (2008), Shahin et al. (2016)
c.443T>C (p.Ile148Thr)	rs35516286	Egyptian	Shahin et al. (2016)
c.464G>A (p.Gly155Asp)	NM_001048174.2	Egyptian	Shahin et al. (2016)
c.3067_3072del (3199del6) (p.Ile1023_Val1024del)	rs121908767	Egyptian	Shahin et al. (2016)
c.1040G>C (p.Arg347Pro)	rs77932196	Egyptian	Shahin et al. (2016)
c.3484C>T (p.Arg1162Ter)	rs74767530	Egyptian	Shahin et al. (2016)
c.1631C>A (p.Ala544Glu)	XP_016873571.1	Egyptian	Shahin et al. (2016)
c.3752G>A (p.Ser1251Asn)	rs74503330	Egyptian	Fathy et al. (2016)
c.57G>A (p.Trp19Ter)	rs397508762	Tunisian	De et al. (2013); Mohamed, (2020)

#### Morocco

**FIGURE 3:**
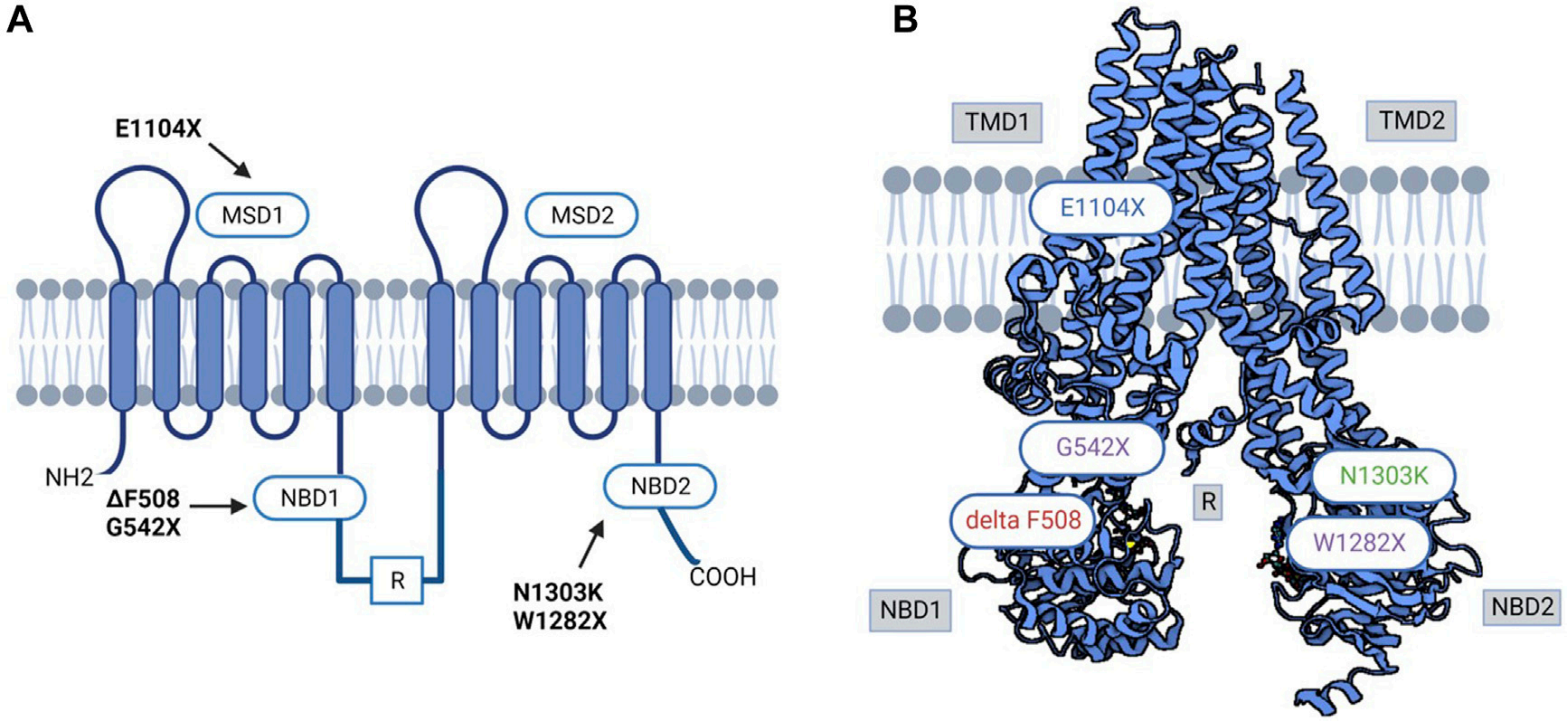
**(A)** 3D and Schematic CFTR structure diagram of prevalent variants in North Africa. **(B)** Variant in red was reported in all North African populations; variants in purple were reported in Tunisian, Algerian, and Egyptian populations; variant in green was reported in Egyptian, Algerian, Tunisian, and Libyan populations. MSD: membrane-spanning domain, TMD: transmembrane domain, NBD: Nucleotide-binding domain, R: regulatory domain.

In the Moroccan population, the epidemiology of CF is poorly documented, and the percentage of CF carriers in the general Moroccan community has never been studied (Ratbi et al., 2008; Ratbi and Sefiani, 2011). There is limited data about Moroccans with CF who migrated to Europe. To our knowledge, no information on the frequency of CF variants among the native Moroccan population exists. A study included 150 healthy native Moroccans (unfortunately, the exact ethnic background of these Moroccan patients was not determined) who were screened at the Institut National d'Hygiène for 32 *CFTR* gene variants. Two people were heterozygous for the p.Phe508del variant, and eight others were heterozygous for the 5T (c.1210-12T[5]) variant (Ratbi et al., 2008) (Figure 3A, B).

#### Algeria

Like Morocco, individuals in Algeria are also at risk of CF; however, there is a lack of information and data in the literature about the incidence, clinical profile, and range of *CFTR* gene variants. A variety of studies have been performed (Loumi et al., 1999; Loumi et al., 2008; Sediki et al., 2014) in Algeria on CF patients, where 27 exons of the *CFTR* gene were screened, and scanning the 30 variants that are most prevalent in the Northern European population was performed in these studies. The following variants were identified in the 27 exons screened: c.3909C>G (p.Asn1303Lys), c.579 + 1G>T (711 + 1G>T), c.2260G>A (p.Val754Met), c.1680-1G>A (1812−1G→A), c.2051_2052delinG (2183AA/G), c.1624G>T (p.Gly542Ter), c.1684G>A (p.Val562Ile), c.1477_1478del (p.Gln493fs), c.4139delC (p.Thr1380AsnfsX4), c.3846G>A (p.Trp1282Ter), c.2930C>T (p.Ser977Phe), CFTRdel2-3 (21 Kb) (Loumi et al., 2008). In addition, screening of the 30 most common variants in the Northern European population revealed the following results: c.1521_1523delCTT (p.Phe508del), c.579 + 1G>T (711 + 1G>T), c.1624G>T (p.Gly542Ter), c.3909C>G (p.Asn1303Lys), and c.1652G>A (p.(Gly551Asp) (Sediki et al., 2014) (Figure 3A, B).

#### Egypt

In a centralized study of 60 patients admitted for CF clinically diagnosed with two positive sweat tests, patients were tested for the 36 most frequent variants. The 36 variants examined were present in 27 out of the 60 patients. The authors, however, did not identify all 36 variants screened for, but they did identify frequent and rare variants in the Egyptian population with CF. Among the 120 alleles studied, 50 positive alleles (41.6%) were detected. Among these, c.1521_1523delCTT (p.Phe508del) represented 58%, followed by c.2051_2052delinG (2183AA/G) (10%). Testing of intron 8 (T) n variations showed that the T7 (c.1210-12T[7]) allele (71.7%), T9 (c.1210-12T[9]) allele (25.8%), and T5 (c.1210-12T[5]) allele (2.5%) were the three most common alleles. The variants detected in this study were c.1521_1523delCTT (p.Phe508del) (58%), c.2051_2052delinG (2183AA/G) (10%), c.3909C>G (p.Asn1303Lys) (6%), c.443T>C (p.Ile148Thr) (4%), c.3846G>A (p.Trp1282Ter) (4%), c.464G>A (p.Gly155Asp) (2%), CFTRdel2-3 (21 KB) (2%), c.3067_3072del (3199del6) (2%), and c.1040G>C (p.Arg347Pro) (2%). Moreover, patients who were homozygous for p.Phe508del reported having more severe clinical presentation than other patients harbouring different variants or compound heterozygosity. The variant 2183AA/G (a frameshift variant A to G at 2183 and deletion of A at 2184), reported to be related to the moderate-severe form, was encountered in 10% of this studied population. Additionally, this variant was found to exist at the same rate in other populations in Syria and Algeria, and it was encountered at a similar frequency (Loumi et al., 2008; Jarjour et al., 2018), and it was identified additionally in Iran, Latin America, and Southern Europe (Rolfini and Cabrini, 1993). In addition, two variants were reported only in the Egyptian population: c.3484C>T (p.Arg1162Ter) and c.1631C>A (p.Ala544Glu), in a 6% and 4% ratio, respectively. Out of 27 patients in the reported study, 16 were homozygous, 7 were compound heterozygous, and 4 were heterozygous. Furthermore, 57% of patients were consanguineous, with a CF family history in 23% (Shahin et al., 2016). The same centre conducted another study for another 100 patients who presented respiratory symptoms; in this study, out of the 100 cases, only 36 tested sweat positive; genetic tests were performed on them, where 10 patients were negative (27%), 14 were heterozygous (38%), and 8 were homozygous (22%) (El-Falaki et al., 2014). Overall, both studies concluded that c.1521_1523del (p.Phe508del) was present in approximately 42% of homozygous cases, and approximately 27% were heterozygous. However, the *CFTR* gene was not completely sequenced, and the inclusion criteria could over- or underestimate many variants, considering that the clinical presentation is reported to be more clinically severe than for other variants. An extensive study, including middle clinical presentation, could shed more light on the mutational spectrum in Egyptian patients. These observations could be made from another study conducted by Al-Haggar et al. that included patients with difficult-to-treat asthma. In this study, the analysis of the CFTR gene found that out of the 61 patients, 20 were heterozygous, and 4 were homozygous for p.Phe508del. In addition, the second most represented variant was c.3846G>A (p.Trp1282Ter), with 25 patients harbouring the variant, of which 20 were compound heterozygous. In comparison, for the remaining 5 patients, the study could not determine the second implicated variants. p.Phe508del/p.Gly542Ter was found in 12 patients, and consanguinity was 70% in this study cohort. Six carriers of single variants in this research had minor respiratory symptoms and negative sweat tests. Out of the 112 probands of difficult-to-treat asthma, 61 individuals carried one or more of the studied three variants (54.5%): 36 individuals had two variants (considered CF after sweat chloride testing for confirmation) and 25 individuals carried single variants (Al-Haggar et al., 2020) (Figures 3A,B).

#### Libya

There is little information about CF in Libya. To date, only ten unrelated Libyan families with CF children have been the subject of a single study, which revealed four variants (c.1521_1523del (p.Phe508del), c.1670delC (p.Ser557fs), c.3909C>G (p.Asn1303Lys), and c.3310G>T (p.Glu1104Ter)), with p.Glu1104Ter having the highest incidence (Hadj Fredj et al., 2011). This is probably because Libya’s first CF centre was only opened in 2008. The literature states that 31 individuals were diagnosed with CF from the CF centre’s creation date till December 2010 (Repetto et al., 2011) (Figure 3A, B).

#### Tunisia

Although North African countries, including Tunisia, account for a significant majority of CF reports and statistics in the African continent (reviewed in (Abubakar Bobbo et al., 2023)) and while clinical testing for CF has been conducted in Tunisia since the 1990s, global frequency and incidence of CF in Tunisia is still poorly estimated, and the genetic background is not yet fully deciphered. Considered a rare disease with variable clinical manifestations, CF may still be underdiagnosed due to the restricted access to the sweat test, especially in public healthcare centres, the limited awareness about the disease pathophysiology by both medical professionals and patient’s families, mainly in the rural areas and the limited number of specialized genetic testing/counselling professionals. The first report on the genetics of CF in the Tunisian population was published by Messaoud et al. and dates back to 1996 (Messaoud et al., 1996). Subsequently, numerous CF cases have been identified thanks to the improvement of diagnostic tools (FREDJ et al., 2013; Boussetta et al., 2018; Hamouda et al., 2020). A molecular study on *CFTR* gene coding region analysis in CF Tunisian families revealed twelve variants (c.1521_1523del (p.Phe508del), c.3310G>T (p.Glu1104Ter), c.3909C>G (p.Asn1303Lys), c.579 + 1G>T (711+ 1T > G), c.3846G>A (p.Trp1282Ter), c.1624G>T (p.Gly542Ter), c.3472C>T (p.Arg1158Ter), c.3889dup (4016insT), and c.2353C>T (p.Arg785Ter), c.3067_3072del (p.Ile1203Val), c.1679 + 5A>G (1811+5A > G), and c.4136 + 2T>G (4268 + 2T > G)). With p.Phe508del being the most common variant in the Tunisian population, which is higher than in other North African populations. According to the literature, this variant may have been imported into the country at the time of the Roman and Vandal invasions. The p.Glu1104Ter variant is this population’s second most common variant (Fredj et al., 2009). Two case reports revealed the presence of the rare c.57G>A (p.Trp19Ter) variant. This nonsense variant has been described thus far in the Tunisian population, suggesting that c.57G>A (p.Trp19Ter) is specific to Tunisian CF patients with significant morbidities (De et al., 2013; Mohamed, 2020). A third case report by Hadj Fredj and coworkers identified a new variant c.3598_3599delinsG (p.Lys1200fs), in exon 19 of the *CFTR* gene in combination with 3442 G→T (p.Glu1104Ter) variant in a Tunisian CF patient (FREDJ et al., 2013). More recently, the same variant was identified again by Hamouda et al., who have published a preliminary national report on CF’s clinical and genetic characteristics in Tunisia. This study involved 123 Tunisian children with CF from the country’s north, center, and south (Hamouda et al., 2020). The genetic screening of the *CFTR* gene resulted in the identification of 17 variants, including six novel variants (c.254G>A (p.Gly85Glu), c.1392 + 6_1392+7insA (1524 + 5 InsC), c.2290C>T (p.Arg764Ter), c.1545_1546delTA (1677delTA), del ex1-2, c.3196C>T (p.Arg1066Cys)) with p.Phe508del being again the most prevalent variant followed by three other variants (c.1624G>T (p.Gly542Ter), c.3846G>A (p.Trp1282Ter) and c.3909C>G (p.Asn1303Lys)) commonly identified in the Mediterranean area (Figure 3A, B). Interestingly, the available data points to the fact that CF is probably more prevalent in the south than elsewhere in the country and that the homozygous form is predominating for almost all identified variants. This is in line with the higher consanguinity rates reported in these regions that potentially increase the likelihood of inheriting autosomal recessive disorders like CF. Moreover, it seems that the distribution of *CFTR* variants in the Tunisian population is different compared to the rest of the populations in North African countries. Particularly, unique variants such as c.2766del8 (p.Leu878PhefsX15), c.3497T>G (p.Phe1166Cys), and c.3128T>G (p.Leu1043Arg) have been exclusively identified, pinpointing their specificity to the Tunisian population (Messaoud et al., 1996; Boussetta et al., 2018). Confirming this specificity by larger scale genetic studies might be very helpful not only from the anthropological side but also from the medical side knowing that this will facilitate designing personalized *CFTR* variant modulators and CF therapeutic tools.

### The way forward (NBS program, NGS, treatment with modulators)

Cystic fibrosis is recognized as a rare disease, yet it is becoming increasingly important for public health organisations at national and international levels. In Africa, people with rare diseases must combine their specific needs with more fundamental requirements, such as nutrition and preventing infectious diseases (Baynam et al., 2020). The development of newborn screening programs (NBS), formalized airway-clearing therapy, and reduced malnutrition by the use of efficient pancreatic enzyme replacement and a high-energy, high-protein diet are just a few examples of the many approaches to how clinical care has improved (Bell et al., 2020b). In nations with well-developed CF research, CF registries, and NBS, the median survival rates for CF patients have risen significantly over the years. In Canada, it is 52 years; in the United States, it is 42; in Europe, it is 40; and in Australia, it is 27 (Van Rensburg et al., 2018). Compared to African countries with underdeveloped CF research, patient’s life expectancy is lower. Based on data from 2008, a CF patient’s life expectancy in South Africa (SA) was less than 21 years. (Van Rensburg et al., 2018). Diagnosing within the first few weeks of birth is too late to achieve the best outcomes. If CF remains undiagnosed and untreated early in life, it can progress to severe symptoms (De Boeck, 2020). Based on CF registry data, it has been expected that each patient whose diagnosis is delayed will spend approximately one million euros more on treatment during their lifetime than a patient detected through NBS (Stewart and Pepper, 2016). Based on these observations, CF newborn screening has been implemented in several nations. We now understand that CF newborn screening improves survival, and its advantages outweigh its risks (De Boeck, 2020).

In addition, the discovery of the *CFTR* gene significantly improved CF diagnosis and treatment. This discovery has increased the capacity to diagnose CF and genotype patients concurrently, identify pancreatic functional status immediately, and plan therapeutic strategies including CFTR modulator selection based on the genotypes. The past 10 years have seen an increase in the use of next-generation sequencing (NGS) methodologies for genetic and genomic sequencing. According to reports, NGS has successfully used DNA isolated from commonly dried blood spot specimens to improve the detection capacity of *CFTR* pathogenic variants. All of the coding areas, intron/exon borders, and chosen intronic sections were designed to be sequenced by the NGS assay (Farrell et al., 2020). In South Africa, NGS is becoming more widely available and less expensive. This diagnostic approach presents, however, difficulties related to the interpretation of the clinical significance of detected variants, many of which can be predicted to be novel in the SA population. Research is now being conducted to determine the best and most economical method and strategy for molecular confirmation of CF in SA (Zampoli On Behalf Of The Msac M, 2018). Access to NGS and other molecular tests is still limited in numerous African countries, including those in North Africa. This limitation is due to various challenges, including the scarcity of adequate facilities, lack of sufficient funding, and inefficiencies in health systems. Further complicating the situation is the underrepresentation of African population data in widely used databases, which hinders the ability to obtain accurate molecular diagnoses for individuals in these regions. Consequently, despite South Africa’s advancements in this field, many other African countries continue to struggle with accessing NGS and molecular testing facilities. (Adebamowo et al., 2018; Phillips et al., 2021; Lumaka et al., 2022).

The discovery of innovative small-molecule treatments that target fundamental *CFTR* malfunction on several levels brings CF to the forefront of precision medicine (De Boeck and Amaral, 2016; Quon and Rowe, 2016). Significant positive impacts on outcomes such as pulmonary function, pulmonary exacerbations, and nutrition have been observed in clinical trials with the *CFTR* modulator medications ivacaftor, which targets the p.Gly551Asp variant, and lumacaftor/tezacaftor, which targets the p.Phe508del variant (Elborn et al., 2016; Taylor-Cousar et al., 2017). None of these treatments is now registered or regulated in any African country, and it is unlikely that medical insurance plans or the public health sector will start funding them anytime soon considering the price is currently US$300,000 per year. The Medical and Scientific Advisory Committee (MSAC) and the CF community are interacting with global pharma to push for accessible pricing for these new medications (Zampoli On Behalf Of The Msac M, 2018).

## Conclusion

In this review article, we gathered available data about CF’s pathophysiology, prevalence and genetic backgrounds in North African populations. We discussed herein the lack and scarcity of CF epidemiological data in these populations, pointing out additionally the challenges impeding proper management of the disease, particularly the limited access to diagnosis tools (sweat tests, genetic testing counselling, etc.), which in turn lead to CF misdiagnosis and underdiagnosis in these countries.

The prevalence of CF phenocopy diseases and limited access to specialized healthcare made it difficult for many African children with CF to be correctly diagnosed, preventing them from receiving adequate medical follow-up (Stewart and Pepper, 2017). Additionally, consanguineous marriages are common in Arab and North African countries. For instance, in Tunisia and Morocco, consanguineous marriages account for 40%–49% and 29%–33% of all unions, respectively. This cultural practice leads to more children born with congenital malformations, recessive diseases, and increased morbidity and mortality (Jaouad et al., 2009; Anwar et al., 2014). Patients with CF born in Africa face a higher risk and can expect to live approximately half as long as their European counterparts. The absence of an effective and comprehensive public health strategy for CF in African countries is mainly responsible for this disparity (Stewart and Pepper, 2016). Large-scale genetic and epidemiological studies focusing on African individuals displaying CF symptoms are crucial for enhancing CF screening yield in the region.

In the presence of several prevalent CF phenocopic diseases, such as recurrent pulmonary infections and PEM and with the complexity of CF as a multisystemic disease, it became more and more apparent that relying only on the clinical investigation is not sufficient to confirm or rule out CF in African patients. We propose that now is the right time to transition towards genomic sequencing as the primary diagnostic method. Integrating NGS with the appropriate bioinformatics pipeline could significantly reduce the time and cost associated with CF diagnosis (Stewart and Pepper, 2017). Our group is currently developing a method to sequence the *CFTR* gene completely using long-read-based Oxford Nanopore Technologies (El Makhzen et al., 2023). We suggest this approach could be well-suited for genetic laboratories with limited resources since it is cheaper and relatively easy to implement. Nearly all African studies on *CFTR* pathogenic variation in CF patients have been limited only to genetic mutation screening without any further functional investigations. Given this, there is also an increasing need to venture into the functional exploration of population-specific variants in Africa. This would also permit the development of personalized variant modulators and therapeutic tools for thousands of young CF patients. Finally, promoting education about CF in the African healthcare sector will also be an asset that will allow better outreach to CF patients, better selection of CF care practices, and the implementation of a participative approach in which both patients with CF and their families are involved in improvement efforts, as demonstrated by the sponsored North American CF Conference (NACFC), which has resulted in an increased CF patients’s survival in the United States of America (Mogayzel et al., 2013).

## Abbreviations

ABC, ATP-binding cassette; CF, Cystic Fibrosis; CFTR, cystic fibrosis transmembrane conductance regulator; CFTR-RD, CFTR-related disorders; DIOS, distal intestinal obstruction syndrome; MSAC, Medical and Scientific Advisory Committee; MSD1, MSD2, membrane-spanning domains; NBS, newborn screening programs; NGS, next-generation sequencing; PKA, phosphorylation by protein kinases A; PKC, phosphorylation by protein kinases C; PM, plasma membrane; R, regulatory domain.
